# Arthroscopic Findings in Anterior Shoulder Instability

**DOI:** 10.2174/1874325001711010119

**Published:** 2017-02-28

**Authors:** Michael Hantes, Vasilios Raoulis

**Affiliations:** Department of Orthopaedic Surgery and Musculoskeletal Trauma, Faculty of Medicine, School of Health Sciences, University of Thessalia, Larissa, Greece

**Keywords:** Anterior shoulder instability, Bankart operation, Arthroscopic findings, Hill-Sachs lesion

## Abstract

**Background:**

In the last years, basic research and arthroscopic surgery, have improved our understanding of shoulder anatomy and pathology. It is a fact that arthroscopic treatment of shoulder instability has evolved considerably over the past decades. The aim of this paper is to present the variety of pathologies that should be identified and treated during shoulder arthroscopy when dealing with anterior shoulder instability cases.

**Methods:**

A review of the current literature regarding arthroscopic shoulder anatomy, anatomic variants, and arthroscopic findings in anterior shoulder instability, is presented. In addition, correlation of arthroscopic findings with physical examination and advanced imaging (CT and MRI) in order to improve our understanding in anterior shoulder instability pathology is discussed.

**Results:**

Shoulder instability represents a broad spectrum of disease and a thorough understanding of the pathoanatomy is the key for a successful treatment of the unstable shoulder. Patients can have a variety of pathologies concomitant with a traditional Bankart lesion, such as injuries of the glenoid (bony Bankart), injuries of the glenoid labrum, superiorly (SLAP) or anteroinferiorly (*e.g*. anterior labroligamentous periosteal sleeve avulsion, and Perthes), capsular lesions (humeral avulsion of the glenohumeral ligament), and accompanying osseous-cartilage lesions (Hill-Sachs, glenolabral articular disruption). Shoulder arthroscopy allows for a detailed visualization and a dynamic examination of all anatomic structures, identification of pathologic findings, and treatment of all concomitant lesions.

**Conclusion:**

Surgeons must be well prepared and understanding the normal anatomy of the glenohumeral joint, including its anatomic variants to seek for the possible pathologic lesions in anterior shoulder instability during shoulder arthroscopy. Patient selection criteria, improved surgical techniques, and implants available have contributed to the enhancement of clinical and functional outcomes to the point that arthroscopic treatment is considered nowadays the standard of care.

## INTRODUCTION

The glenohumeral joint presents with the greatest range of motion of all joints in the human body, and preservation of its stability is essential to its function [1]. The shoulder joint is an inherently unstable ball and-socket joint, and it is susceptible to a variety of injuries. This joint has complex anatomy and its stability is conferred by a combination of bone, soft tissue and muscular structures. It is therefore the most commonly dislocated joint, with an overall incidence of approximately 24/100.000 per year [2] and over 90% of dislocations are anteriorly displaced [3]. In young patients between 20 and 29 years of age appears the highest incidence of shoulder dislocation [4] and this group of patients has also the highest recurrence rate [5-8]. Traumatic glenohumeral instability is defined as occurring after an inciting event that results in subjective or objective subluxation or dislocation that is reduced either spontaneously or by a health professional [9]. Atraumatic instability occurs as the result of generalized ligamentous laxity or repetitive motion, as in overhead throwing athletes. Inferior and multidirectional instabilities are less common than anterior and posterior ones and have been described to combine the presence of a sulcus sign or inferior subluxation of the humeral head with symptoms of pain or instability [10, 11]. Anterior shoulder instability accounts for 95% of acute traumatic dislocations. Although there are patients who suffer an initial shoulder dislocation and never experience a second episode of shoulder instability [12] a significant percentage present with recurrent instability that results in morbidity and decreased functionality, in respect to the demands placed on the joint during every day, occupational and athletic activities [12].

Recurance of anterior shoulder instability has been correlated with a younger age at the time of first dislocation. Among 255 patients with primary traumatic anterior dislocation, who were treated with a sling for 4 weeks, the recurrence rate was 55% 2 years after the initial traumatic dislocation. In addition, 66% of the patients had an episode of instability within 5 years [12]. In Burkhead *et al*. study, 324 shoulders were followed for at least 10 years after primary anterior dislocation [13]. Ninety-four percent of the patients younger than 20 years had a recurrence compared with 14% of those older than 40 years. The patients without shoulder immobilization had a 70% recurrence rate, while this percentage, decreased to 26% and to 46% when immobilized for 1 to 3 weeks respectively [13]. These findings suggest that younger patients with primary anterior dislocations have a significantly higher rate of recurrence. The effectiveness of rehabilitation is still in debate. Burkhead & Rockwood evaluated the effect of rehabilitation in 115 patients, with traumatic and atraumatic recurrent shoulder subluxation, who underwent a muscle-strengthening exercise regimen [14]. As a result, 16% of the shoulders with traumatic etiology had excellent or good results in contrast to 80% of those with atraumatic etiology. The authors highlighted the importance of identifying the etiology of instability to ascertain a successful result out of conservative treatment. In a prospective randomized clinical trial, active patients aged less than 30 years who were treated with supervised physical therapy showed recurrence rates of 17 to 96% whereas arthroscopic instability repair had failure rates between 4% and 22% [15]. These findings indicate that young, highly active patients would benefit from early, arthroscopic repair after first-time traumatic anterior shoulder dislocation, compared with conventional non-operative treatment.

The treatment of shoulder instability begins with a thorough preoperative history and physical examination, followed by careful evaluation, interpretation, and clinical correlation of imaging studies. Once the etiologic factors contributing to instability are identified, it is imperative to determine the presence or absence of co-pathologies. This is especially important preoperatively (to plan for specific repair strategies) and intra-operatively, to prevent otherwise avoidable postoperative complications. Arthroscopic shoulder stabilization is the treatment of choice in cases of recurrent instability [16, 17]. The outcome is usually excellent when performed by experienced surgeons, and additional procedures may be performed to address concomitant pathologies such as a SLAP lesion or a wide rotator interval. For optimal outcome, the surgeon must be well aware of the normal anatomy, the various normal anatomic variants (*e.g*., sublabral hole, Buford complex, and meniscoid labrum), and the various pathologic presentations. This distinction is especially challenging, as shown by a recent study which highlighted that, the inter-rater reliability for the exact description of anatomic structures such as the inferior glenohumeral ligament (IGHL) or the bony glenoid size is below 40% during shoulder arthroscopy [18]. The will of surgeons to identify any associated injuries predisposing to recurrent instability, has resulted in a threefold increase in the use of magnetic resonance imaging (MRI) [19] and in the use of magnetic resonance arthography (MRA). MRA has a high sensitivity when used to identify associated injuries in shoulder dislocation, although in 13% of the cases an additional injury was identified during arthroscopy [20].

Patients can have a variety of pathologies concomitant with a traditional Bankart lesion. If all pathology is not recognized and treated appropriately, the patients’ shoulder instability, pain, and diminished function may persist. Surgeons must be well prepared and understanding the normal anatomy of the glenohumeral joint, including its anatomic variants, to seek for the possible pathologic lesions in anterior shoulder instability during shoulder arthroscopy. Therefore the aim of this review is to focus on the possible arthroscopic findings in anterior shoulder instability.

## ANATOMY AND ANATOMIC VARIANTS

Stability of the glenohumeral joint, is produced by static structures and dynamic stabilizers. Static stabilizers include the bony anatomy, labrum, capsule, glenohumeral ligaments and rotator interval. Dynamic stabilizers include the rotator cuff, long head of the biceps, the deltoid and scapular muscles. The “suction cup effect” (negative pressure within the joint) also contributes to stability, which helps center the humeral head independently of muscular forces and is primarily important in the midrange, where the capsule and ligaments are not under tension. The bony anatomy of the glenohumeral joint also plays a significant role in stability. The glenoid is more concave in the superoinferior than the anteroposterior direction. In addition, the articular cartilage is thicker towards the periphery of the glenoid, thus increasing the depth of the concavity. Because the size of the glenoid is limited compared with the humeral head, even a relatively small bone loss of the glenoid, may reduce considerably the surface area for articulation and consequently compromise stability. A bone loss that exceeds 20% of the glenoid surface is considered critical for the recurrence of instability [21, 22]. The labrum is a fibrocartilaginous structure attached to the glenoid rim. It functions, to increase the antero-posterior and supero-inferior depth of the glenoid and the surface contact area for the humeral head. Specifically, it increases the concavity of the glenoid up, to 9mm in the superior-inferior direction and the antero-posterior depth to 5mm [23]. Labral resection reduces resistance to translation by 20% [24]. The labrum also provides an attachment site for the glenohumeral ligaments. Two types of labral attachments to the glenoid have been described. The first is around the periphery through a fibrocartilaginous transition zone, which creates mobility along the central border similar to the knee meniscus. The second is securely attached both peripherally and centrally. The anteroinferior attachment of the labrum to the glenoid is normally tight. On the contrary, the superior attachment inserts directly into the biceps tendon distal to the insertion on the supraglenoid tubercle, it is loose and anatomically variant. Isolated lesions of the superior labrum do not result in instability. However, if the biceps insertion is also destabilized, significant translation occurs [25]. The glenohumeral capsuloligamentous system provides a restraint to excessive translation in varying positions of the joint. In particular, the anterior band of the inferior glenohumeral ligament (AIGHL) attaches to the anteroinferior labrum and primarily resists anteroinferior translation in the abducted externally rotated shoulder position. The rotator cuff compresses the humeral head into the glenoid throughout the range of motion. An association between undersurface rotator cuff tears and instability has been described [26]. The rotator interval (RI), which lies between the leading edge of the supraspinatus and the superior edge of the subscapularis, has also been implicated in glenohumeral instability. Closure of a large defect in the RI has been shown to decrease inferior instability. There may be an inverse relationship between the size of the RI and the superior glenohumeral ligament (SGHL) contributing to the instability [27].

The normal variations of the glenoid labrum are small recesses or a meniscoid (like a small meniscus) appearance, in the superior and anteriorsuperior regions. Antero-superiorly, the sublabral hole may be found. It is defined by the complete separation of the labrum from the glenoid in this region. The incidence of the sublabral hole varies between 9% and 17.5% [28, 29]. The Buford complex, in which the anterior-superior labrum is absent and replaced by a cordlike middle glenohumeral ligament (MGHL), can be encountered and mistaken for a separation of the anterior labrum. The incidence of the Buford complex varies between 1.5% and 6% [30, 31]. In shoulders with a Buford complex, the superior glenohumeral ligament (SGHL) may be absent, whereas the IGHL is usually well developed. In shoulders with significant SLAP lesions (described later), the incidence of a sublabral hole increases to 40% and the incidence of a Buford complex increases to 20%. Whether these anatomic variants place increased load on the biceps anchor is not known.

In about 80% of cases it is described in the anterior-superior part, a subscapular recess (subscapular bursa, Weitbrecht foramen), which is an opening of the anterior capsule into the sub-tendinous bursa of the subscapularis muscle and is mostly located between the SGHL, the subscapularis tendon, and the MGHL [32]. DePalma *et al*. describe 6 different variants of this recess [33]. The variations of the glenohumeral ligaments according to Morgan *et al*. are: a) SGHL, MGHL, and IGHL can be separately identified in 66% of cases, b) IGHL and MGHL are fused in 7% of cases, c) MGHL has a strong cordlike shape in 19% cases, and d) all ligaments missing completely in 8% cases [34]. Sometimes a cordlike variant of the MGHL can be found and in other cases the MGHL is often missing or indistinct in patients with anteroinferior shoulder instability. It is considered that the presence of a cordlike MGHL is regarded as a protective factor against shoulder instability [28].

## IMAGING

**Fig. (1):**
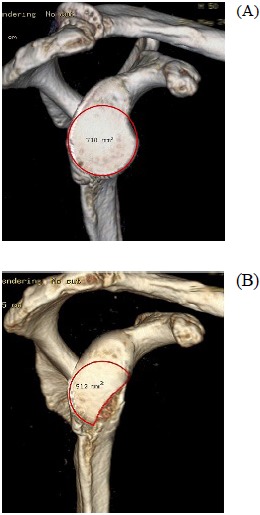
(**A**, **B**). Volume-rendering 3D reconstructed image of a cadaveric shoulder before (**A**) and after (**B**) artificially creating a glenoid bone defect. The surface area of the inferior glenoid is being measured.

Routine radiographic imaging of the shoulder should include a true anteroposterior, axillary and scapular-Y views. Hill-Sachs lesions can be best appreciated on the anteroposterior view in internal rotation and the notch view [35]. Velpeau or West point axillary views can be used to visualize avulsion fractures and glenoid bone deficiencies [36]. Advanced imaging has offered an improved ability to evaluate soft tissue lesions as well as glenohumeral deficiencies following shoulder dislocation. MRI has become the gold standard in evaluating glenohumeral instability demonstrating a high accuracy for detecting labral tears using non-contrast, enhanced imaging techniques [37]. MR arthrography however, has been found to present the highest sensitivity in detecting labral pathology compared with plain MRI and CT arthrography [38, 39]. It also achieves the best visualization of the inferior glenohumeral ligament and labrum. Both MRI and MR arthrography can also be helpful in evaluating bone loss. However, recently volume-rendering three-dimensional CT scans have offered a highly accurate method of measuring glenoid deficiencies and Hill-Sachs lesions. Jordan *et al*. described a failure of MRA to demonstrate associated injuries in 13% (soft-tissue Bankart 5%, SLAP 5% and osseous Bankart lesions 3%). These findings suggest that CT scans should be considered if further clarification of on osseous injury is required [39]. With CT the humeral head can be digitally subtracted to allow for preoperative measurement of the inferior glenoid surface and the percentage of bone missing (Fig. **1A**, **B**). Glenoid bone defects, occur along a line parallel to its long axis. The inferior two thirds of the glenoid, have been described as a well-conserved circle and the amount of bone missing is assessed in respect to surface area loss of the circle. Glenoid bone loss of between 6 to 8 mm of the anteroposterior diameter corresponds to 20- 25% of the surface of the inferior glenoid. In a similar fashion, the extent and morphology of a Hill-Sachs lesion, can be evaluated to assess the degree of engagement.

## EXAMINATION UNDER ANESTHESIA

Examination under anesthesia is usually performed to confirm preoperative clinical findings and to assess the degree of instability. A high correlation between arthroscopic findings and examination under anesthesia has been reported [40]. The examination should be performed either in the supine or beach chair positions. Passive range of motion is recorded first with the arm at the side and 90° of abduction. With the arm abducted at 90°, posterior and anterior forces are applied to provoke translation of the humeral head in relation to the glenoid. Laxity is classified as grade 1 (translation to the glenoid rim), grade 2 (translation over the glenoid rim with spontaneous reduction) and grade 3 (dislocation without spontaneous reduction). By applying an inferior force to the adducted arm the sulcus sign is elicited. A sulcus sign is tested in adduction and external rotation, and also at 45° abduction that tightens the inferior capsule. A persistent sulcus sign in adduction and external rotation is indicative of rotator interval pathology. Neither of these can be easily achieved without comparison to the asymptomatic side, and hence no examination under anesthesia is complete without examination of both shoulders.

## PATIENT POSITIONING

**Fig. (2):**
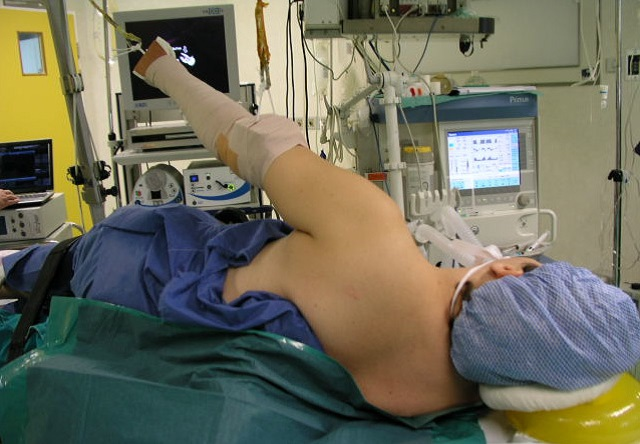
Lateral decubitus setup for arthroscopic instability repair. Axial and lateral traction is applied with the arm at approximately 45° abduction.

The patient can be positioned in either the lateral decubitus or beach chair positions, which is mainly based on surgeon preference. The beach chair position affords several advantages, including the ease to address concomitant rotator cuff pathology and the ability to convert to open surgery if necessary. However, the authors’ choice is to use the lateral decubitus position as it is easier to address the pathology at the anteroinferior capsulolabral complex. The arm is usually placed at 45° abduction and traction is applied both in the axial and lateral directions (Fig. **2**). One of the disadvantages of this patient setup is the difficulty to achieve rotational control during the repair. For example, subscapularis repair and rotator interval closure are best performed in 30° to 45° of external rotation, which cannot be easily done at the lateral position.

## INSTRUMENTATION

Basic equipment for shoulder arthroscopy includes a tower containing a video monitor, control box, light source, shaver power and electrocautery source, and irrigation pump. A 30-degree arthroscope is usually adequate for most arthroscopic procedures in the shoulder. Fluid pressure within the joint should be kept around 30mmHg and may increase up to 70mm Hg for viewing the subacromial space. Maintaining a systolic arterial pressure below 100mm Hg improves visualization. Increased fluid pressure or flow, may cause extravasation of fluid into the surrounding soft tissue, distort the anatomy intra-operatively and increase morbidity postoperatively

## ARTHROSCOPIC PORTALS

**Fig. (3):**
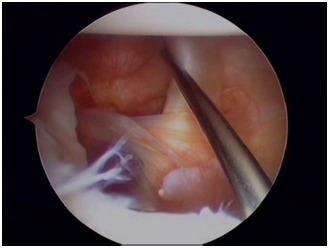
The anterior-inferior portal is created, just above the superior edge of the subscapularis.

**Fig. (4):**
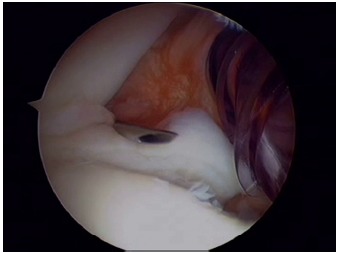
The anterior-superior portal is then created between the biceps tendon (needle) and the subscapularis.

**Fig. (5):**
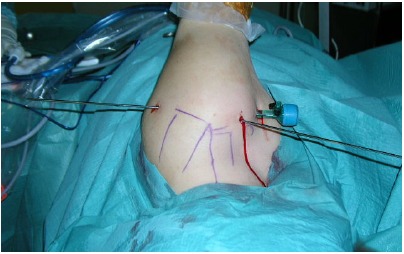
Typical posterior, anterior-superior and anterior-inferior portals for arthroscopic anterior instability repair. A working cannula is inserted in the anterior-inferior and Wissinger rods in the remaining two portals.

Usually three portals (two anterior and one posterior) are sufficient for arthroscopic anterior stabilization procedures. Initially, a standard posterior portal is used for diagnostic arthroscopy. It can be created in line with the lateral edge of the acromion and 1cm inferior to its posterior tip to have an improved trajectory in relation to the glenoid. This portal is used for diagnostic glenohumeral arthroscopy and to localize the pathology to be addressed. An anterior-inferior portal is created with an outside-in technique, just above the superior edge of the subscapularis to allow for inferior placement of suture anchors on the lower aspect of the glenoid neck (Fig. **3**). Next, an anterior-superior portal is then created with an inside-out or outside-in technique between the biceps tendon and superior edge of the subscapularis (Fig. **4**). This portal is used for mobilization of the capsulolabral complex and for subsequent suture management. It is always advisable to assess the intra-articular pathology through the anterior-superior portal to better evaluate the extent of labral tear posteriorly or glenoid bone loss and avoid missing a possible ALPSA lesion. Both anterior portals are created within the rotator interval and there should ideally be enough skin bridge between them (2-3cm) to allow for easier handling of arthroscopic instruments (Fig. **5**). Alternative portals have been described, such as a transubscapularis portal described by Davidson and Tibone or a 7’o clock posteroinferior portal for accessing the most inferior aspect of the glenoid. Working cannulas are inserted into the two working portals to facilitate instrumentation handling. A wider (8mm) cannula is preferable for the anterior-inferior portal to allow for curved suture hooks, while a 5.5mm cannula is adequate for the superior portal for grasping instruments to be inserted.

## ARTHROSCOPIC FINDINGS IN ANTERIOR INSTABILITY

Assessment of the mobility of the capsuloligamentous complex is crucial to determine if the soft tissues have been displaced or are scarred in a medial position on the neck of the glenoid. A combination of probes, rasps, motorized shavers and periosteal elevators are used to mobilize the medially displaced soft tissues from the glenoid neck. Care must be taken not to debride normal tissue needed for the repair. During this step, the subscapularis muscle must be visualized underneath the mobilized labral tissue. It is recommended to release tissue, inferiorly to the 6 o’clock position on the glenoid face for optimal mobilization. Attention is then turned towards the glenoid. An abrader or rasp is used to decorticate the glenoid edge while preserving the bone stock. It is important to ensure that the soft tissue remnants have been removed and there is a bleeding bed of bone at the repair site to enhance healing.

**Fig. (6):**
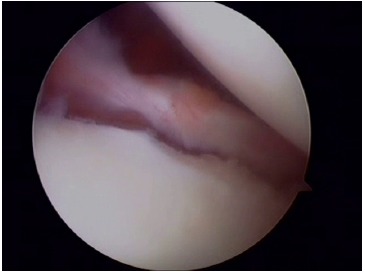
A typical Bankart lesion with detachment of the anterior labrum.

**Fig. (7):**
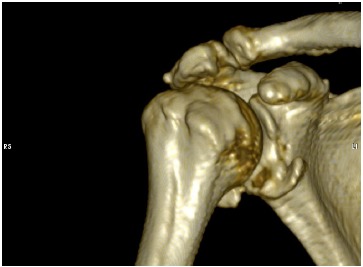
A three-dimensional reconstruction CT-image demonstrating a bony-Bankart lesion.

The commonest sequel of an anterior dislocation and the main cause of instability is the Bankart lesion (Fig. **6**). It is defined as a labral-complex avulsion from the scapular periosteum. The condition was named for British surgeon Arthur Sydney Blundell Bankart, who first describe it. It usually, includes some degree of capsular stretch and injury. Bankart lesion, allows the humeral head to protrude forward from the glenoid cavity, which not only causes the fibrocartilaginous glenoid labrum tear from the front-bottom portion of the glenoid, but also causes the avulsion of the scapular periosteum along the front of the scapular neck. It has been reported that Bankart lesions are responsible for 73-90% of the traumatic recurrent shoulder dislocations [41-43]. When the lesion involves a fracture of the antero-inferior glenoid rim in addition to the soft tissue avulsion it is referred to as bony Bankart (Fig. **7**). Glenoid bone defect is considered one of the major causes of the recurrent dislocation of the shoulder, and in turn, the recurrent shoulder dislocation can further damage the bony structure in the shoulder joint.

**Fig. (8):**
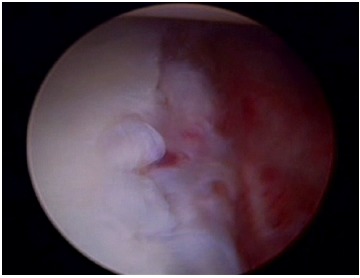
An ALPSA lesion as seen from the anterosuperior arthroscopic portal. The labrum and periosteum have been avulsed and displaced medially.

Anterior labral periosteal sleeve avulsion (ALPSA) is a soft-tissue or bony Bankart lesion that has healed in a medially displaced position on the glenoid rim and therefore, does not restrain adequately the anterior translation of the humeral head (Fig. **8**). In this case, the avulsed periosteum, causes medial and inferior displacement of the labroligamentous structures [44].

**Fig. (9):**
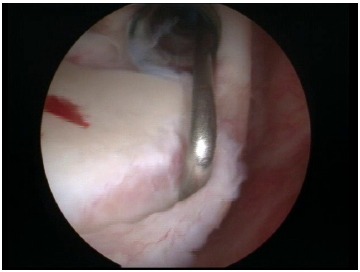
A type II SLAP lesion as seen during arthroscopy.

A superior labrum anterior posterior (SLAP) lesion includes a spectrum of pathologic conditions of the superior labrum that may extend to the biceps root (Fig. **9**). Classification of these lesions was extended by Maffet to include 7 subtypes [45].

Type 1 Fraying of the anterosuperior labrum
Type 2 Superior labrum-biceps complex detachment from glenoid rim
Type 3 Bucket-handle tear of the labrum with an intact biceps anchor
Type 4 Bucket-handle tear of the labrum with detachment of the biceps complex
Type 5 Bankart lesion continues superiorly and includes separation of the biceps complex
Type 6 Unstable flap tear of the labrum with an unstable biceps complex insertion
Type 7 Labrum-biceps complex separation extending beneath the middle glenohumeral ligament

**Fig. (10):**
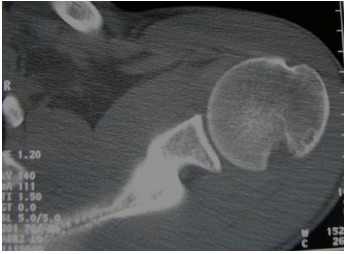
A transverse plane CT image demonstrating a large Hill-Sachs lesion of the humeral head.

**Fig. (11):**
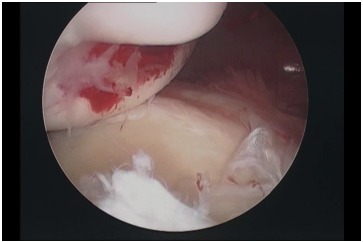
Arthroscopic appearance of a Hill-Sachs lesion.

A Hill-Sachs lesion is an compression fracture at the posterosuperior aspect of the humeral head that results from its impact on the glenoid rim when the humeral head dislocates anteriorly (Figs. **10** and **11**). They occur at 47 to 80% of anterior dislocations and in almost all cases of recurrent instability. If the postero-lateral humeral head engages the anterior glenoid when abducted and externally rotated, the Hill-Sachs lesion is defined as engaging [21]. The size and location of the defect mainly determine the likelihood of engagement. Although usually insignificant, in patients with glenoid bone loss a Hill-Sachs lesion can become more significant and engage the glenoid with much less force and anterior translation than those without glenoid bone loss.

Humeral avulsion of glenohumeral ligaments (HAGL) occurs when the capsuloligamentous structures are avulsed and torn off the humeral head and not the glenoid. An external rotation force in addition to hyperabduction commonly results in this lesion in contrast to a hyperabduction and impaction force that may produce a Bankart lesion [46]. The incidence of HAGL lesions after a traumatic dislocation has been reported at 39% in patients without a Bankart lesion [47]. A bony HAGL lesion occurs when the glenohumeral ligament is avulsed along with a bone fragment of the humeral head [48].

A Perthes lesion is an incomplete avulsion without displacement of the antero-inferior labrum with a medially striped but intact periosteum [49]. It is named after George Perthes an X-Ray diagnostic pioneer who first described the lesion in 1905.

Glenoid labral articular disruption (GLAD) lesion occurs when there is a defect in the articular cartilage of the anteroinferior glenoid in addition to the labral tear, described by Neviaser in 1993 [44]. In these cases, the labrum is not fully detached from the glenoid while there is a glenoid cartilage lesion, and therefore the predominant symptom is these cases, is not instability but pain.

## DISCUSSION

The spectrum of the pathoanatomic lesions encountered in shoulder instability is wide. Yiannakopoulos *et al*. [50] presented that the arthroscopic findings, in patients with acute instability are quantitatively and qualitatively different from the findings in patients with chronic instability. In their study, the incidence of Bankart lesions was 78.2% in patients with an acute dislocation, whereas in chronic cases the incidence of Bankart or ALPSA lesions was 97.11% and this difference was statistically significant. In both groups, the presence of Bankart lesion was recordered in 119 patients (93.7%), chondral or osteochondral Hill-Sachs lesion was noted in 112 patients (88.1%), an ALPSA lesion was found in 13 patients (10.23%). Continuously a SLAP lesion was noted in 26 patients (20.47%), a HAGL lesion was noted in 2 acutely dislocated shoulders (1.57%), and capsular laxity was noted in 33 patients (25.98%). All ALPSA lesions were recordered in patients with chronic instability, whereas both HAGL lesions occurred in patients with acute dislocations. A Hill-Sachs lesion was found in 15 cases in the group with acute dislocations (65.21%) and in 97 cases in the chronic recurrent instability group (93.26%). The capsule was considered lax in 2 patients with acute instability, and in 31 patients with chronic instability (8.69% v 29.8%, P 0.037). According to this study, the incidence of shoulder lesions, increases with time and number of dislocations. Therefore, in symptomatic unstable shoulders, early stabilization may be recommended to prevent secondary injuries.

Weimin Zhu *et al.* [51] inspected thirty-one patients with recurrent anterior shoulder dislocation inspected by arthroscopy. In this study the patients were divided into two groups: 17 with shoulder dislocation and hyper-laxity (the hyperlaxity group) and 14 with only traumatic shoulder dislocation (the trauma group). There was no significant difference in the overall incidence of labral injuries and SLAP lesions between the two groups (P 0.05). However, there was a significant difference in the incidence of Bankart lesions, ALPSA lesions, GLAD lesions, Hill–Sachs lesions, bony defects of the anteroinferior glenoid and rotator cuff injuries between the two groups. Bankart injury occurred more frequently in the trauma group, and ALPSA lesions and glenolabral articular disruption injuries were more common in the hyper-laxity group. Bone or cartilage injury of anteroinferior glenoid was more frequently found in the trauma group.

The knowledge of the variety of associated lesions in shoulder instability is very important and aids to surgical treatment. The most common iatrogenic mistakes in anterior shoulder instability operations leading to instability recurrence, are failure to recognize the presence of a defective anterior glenoid rim and failure to reduce capsular redundancy. The detached anterior labrum, may adhere to the glenoid neck and macroscopically give the impression to the surgeon of a stable connection, which is actually functionally incompetent, forming an ALPSA lesion. Failure to recognize, mobilize, and restore the labrum to its original location is a common cause of failure of arthroscopic techniques. The incidence of ALPSA lesions increases in patients with multiple dislocations [52]. The majority of ALPSA lesions are reported in in patients with chronic instability.

The commonest SLAP lesions in the most large series are the type II tears. On average, 40% of patients with Bankart lesions have an additional type II SLAP lesion [53]. SLAP lesions have been associated with glenohumeral stability. On examination under anesthesia about 43% of patients with SLAP lesions were noted to have increased humeral head translation [54].

The incidence of Hill-Sachs lesion is generally higher than previously thought. A divot or flattening occurs on the articular surface in the posterolateral aspect of the humeral head, posterior to the humeral greater tuberosity and must be distinguished from the denuded articular cartilage, the non-articulating bare area of the humeral head. The Hill-Sachs lesion, may be limited to the articular cartilage or may extend to the subchondral bone. It has been reported that the incidence of Hill-Sachs lesions in acute cases is significantly, less than that in chronic cases [50]. The reported incidence of Bankart and Hill-Sachs lesions varies. Sugaya *et al*. [55] examined 100 patients with recurrent anterior glenohumeral instability and found a Bankart lesion in 97% of their patients and an osseous Bankart lesion in 50%. In another study, where 25 patients with acute shoulder dislocation participated, Bankart and Hill-Sachs lesions were present in 23 cases, whereas loose bodies were noted in 8 cases [56]. In the report of Norlin, in acute shoulder dislocations, the incidence of Bankart and Hill-Sachs lesions was 100% [57].

The diagnosis of a HAGL lesion has been reported in several studies [47, 58-65]. These studies included 71 patients with HAGL lesions. The average age was 27 years, with patients ranging from 12 to 69 years old. There majority of the patients were male, with only 4 female patients in the sample group. Ahigh percentage of the patients (42%) were injured during rugby, while other activities include diving, football, basketball, volleyball, surfing, and skiing as well as some occurring during motor vehicle accidents. In eight patients (11%) a prior orthopaedic surgery of the same injured shoulder have been reported. Sixty-six (93%) of the HAGL lesions were anterior, and the remaining 5 were posterior. Almost two thirds (62%) of the HAGL injuries were associated with concurrent lesions of the shoulder. Eighteen of these were tears of the labrum (25%), 16 were rotator cuff tears (23%), and 12 were Hill-Sachs deformities (17%). HAGL lesions, are an important cause of glenohumeral instability, with a reported incidence of 1% to 9%. The majority of lesions involve the anterior band, and almost two thirds of patients suffer from concurrent injury to the labrum or rotator cuff. In the past undiagnosed or improperly managed HAGL lesions, lead to significant disability in otherwise healthy young individuals.

In atraumatic instability [11] the arthroscopic findings may also be predictable. In a study of 43 patients with atraumatic instability resistant to physiotherapy, 44.2% had capsule elongation, 30.2% had typical Bankart lesions, and 25.6% had complex lesions of the labrum and capsule whereas Hill-Sachs lesions were identified in 60.5%.

## CONCLUSION

Shoulder stability is maintained by the complex interplay between static and dynamic mechanisms. A detailed knowledge of the anatomic structures, including their normal anatomic variations and functional relevance, is appropriate for adequate diagnosis and therapy. The incidence of shoulder lesions increases with time, because the initial dislocation and secondary lesions are more common in patients with chronic instability. Only shoulder arthroscopy allows for a detailed visualization and a dynamic examination of all relevant structures and identification of pathologic findings, such as injuries of the glenoid (bony Bankart, and glenoid defects), injuries of the glenoid labrum superiorly (SLAP) or anteroinferiorly (*e.g*., Bankart, ALPSA, and Perthes), HAGL lesions and Hill-Sachs lesions.

Authors Disclosure statement: We did not receive any funding or sponsorship for this study and we do not have also any other potential financial conflict of interest related to this study.

## LIST OF ABBREVIATIONS

AIGHL: = Anterior band of the Inferior Glenohumeral Ligament
ALPSA: = Anterior Labral Periosteal Sleeve Avulsion
GLAD: = Glenoid Labral Articular Disruption
HAGL: = Humeral Avulsion of Glenohumeral Ligaments
IGHL: = Inferior Glenohumeral Ligament
MRA: = Magnetic Resonance Arthography
MRI: = Magnetic Resonance Imaging
MGHL: = Middle Glenohumeral Ligament
RI: = Rotator Interval
SGHL: = Superior Glenohumeral Ligament
SLAP: = Superior Lesions Anterior to Posterior
